# Detection of acute 3,4-methylenedioxymethamphetamine (MDMA) effects across protocols using automated natural language processing

**DOI:** 10.1038/s41386-020-0620-4

**Published:** 2020-01-24

**Authors:** Carla Agurto, Guillermo A. Cecchi, Raquel Norel, Rachel Ostrand, Matthew Kirkpatrick, Matthew J. Baggott, Margaret C. Wardle, Harriet de Wit, Gillinder Bedi

**Affiliations:** 1 Computational Biology Center – Neuroscience, IBM T.J. Watson Research Center, Yorktown Heights, NY USA; 2 Department of Preventive Medicine, Keck School of Medicine, University of Southern California, Los Angeles, CA USA; 3 Addiction and Pharmacology Research Laboratory, Friends Research Institute, San Francisco, CA USA; 4 Department of Psychology, University of Illinois at Chicago, Chicago, IL USA; 5 Human Behavioral Pharmacology Laboratory, Department of Psychiatry and Behavioral Neuroscience, University of Chicago, Chicago, IL USA; 6 Centre for Youth Mental Health, University of Melbourne, and Orygen National Centre of Excellence in Youth Mental Health, Melbourne, Australia

**Keywords:** Psychology, Human behaviour

## Abstract

The detection of changes in mental states such as those caused by psychoactive drugs relies on clinical assessments that are inherently subjective. Automated speech analysis may represent a novel method to detect objective markers, which could help improve the characterization of these mental states. In this study, we employed computer-extracted speech features from multiple domains (acoustic, semantic, and psycholinguistic) to assess mental states after controlled administration of 3,4-methylenedioxymethamphetamine (MDMA) and intranasal oxytocin. The training/validation set comprised within-participants data from 31 healthy adults who, over four sessions, were administered MDMA (0.75, 1.5 mg/kg), oxytocin (20 IU), and placebo in randomized, double-blind fashion. Participants completed two 5-min speech tasks during peak drug effects. Analyses included group-level comparisons of drug conditions and estimation of classification at the individual level within this dataset and on two independent datasets. Promising classification results were obtained to detect drug conditions, achieving cross-validated accuracies of up to 87% in training/validation and 92% in the independent datasets, suggesting that the detected patterns of speech variability are associated with drug consumption. Specifically, we found that oxytocin seems to be mostly driven by changes in emotion and prosody, which are mainly captured by acoustic features. In contrast, mental states driven by MDMA consumption appear to manifest in multiple domains of speech. Furthermore, we find that the experimental task has an effect on the speech response within these mental states, which can be attributed to presence or absence of an interaction with another individual. These results represent a proof-of-concept application of the potential of speech to provide an objective measurement of mental states elicited during intoxication.

## Introduction

In recent years, psychiatry researchers have endeavored to identify alternative, objective evaluations to aid subjective clinical assessments and diagnoses [1]. One approach analyzes free speech, a promising data source due to its low cost, easy acquisition, and high reliability. Speech, a universal human phenomenon, represents a rich source of semantic, syntactic, and acoustic data that can be mined for clinically relevant information such as quantifying incoherence in schizophrenic speech [2]. In the past, speech assessment was largely reliant on clinical observation, manual coding, or word counting methods (e.g. see [3]). These approaches, while providing important information, have limitations in objectivity and how comprehensively they can assess this nuanced, complex behavior e.g. acoustic components. As a complement to existing methods, recent rapid developments in computerized natural language processing [4] provide increasingly sophisticated automated methods to quantitatively characterize speech and investigate mental states based on the features extracted. These methods are routinely used in industry for the purpose of speech recognition [5], chatbots and conversation agents [6], and recommender systems [7] among others. Whether they could aid research and practice in psychiatry is only beginning to be explored in the context of simulated psychiatric evaluations (e.g. see [1, 8, 9]) or the analysis of alternative ways of communication such as social media (e.g. see [10–12]).

Research on acute drug effects is one area in which investigation of mental states is paramount. Abused drugs profoundly alter mental states in ways that appear to motivate use [13–15]. Mental state changes due to intoxication are typically assessed using standardized self-report measures of relevant subjective states (e.g. “euphoric”, “high”) repeatedly throughout the drug experience [13, 14]. While such approaches provide valuable information, the sensitivity of standardized scales is limited by the mood descriptors included, which may not capture the effects of emerging drugs. Moreover, self-report scales rely on access to interoceptive experiences, as well as motivation and capacity to accurately report them, factors that may vary systematically with drug effects. Computerized analysis of free speech offers the potential to by-pass some of these limitations, providing a more direct “window into the mind” [16].

Based on this rationale, we conducted two initial investigations employing automated natural language processing to assess mental state alterations due to intoxication. In the first, we investigated whether the semantic content of speech while intoxicated could discriminate between different drugs in a small double-blind placebo-controlled within-participants human laboratory study (*N* = 13, [16]). Volunteers received 3,4-methylenedioxymethamphetamine (MDMA; the main psychoactive constituent in ‘ecstasy’ or ‘molly’; 0.75, 1.5 mg/kg), methamphetamine (20 mg), and placebo before undergoing a 10-min free speech task in which they described people close to them. We measured speech semantic content using Latent Semantic Analysis (LSA), a well-validated automated content assessment method [17]. Specifically, for each speech transcription we extracted LSA values for semantic proximity to several concepts chosen a priori to reflect the apparently usual prosocial effects of MDMA (e.g. *empathy*, *friend*, *rapport,* etc.). We found that speech on MDMA (1.5 or 0.75 mg/kg) was closer to relevant concepts such as *empathy*, *rapport*, *friend*, and *intimacy* than speech on methamphetamine or placebo. Moreover, in cross-validated prediction, speech features differentiated MDMA (1.5 mg/kg) and placebo with 88% accuracy, and MDMA (1.5 mg/kg) and methamphetamine with 84% accuracy [16]. Thus, this preliminary investigation indicated that natural language processing of free speech is capable of capturing behavior information associated with clinical studies findings, such as an increased empathy of an individual due to intoxication with MDMA.

In the second within-participants analysis, 35 volunteers received placebo and MDMA (1.5 mg/kg) across two sessions, administered in a randomized double-blind fashion, prior to a 5-min speech task focused on an important person in the participant’s life [18]. Analyses employed a bag-of-words approach, with classification based on how often individual words appeared in speech transcriptions, without reference to their order or context. A random forest machine learning approach classified speech (placebo vs. MDMA) based on the frequency of word occurrence within transcriptions. This allowed identification of the most important words contributing to the classification of speech on MDMA relative to placebo. Words contributing to the classification included some with social content (*outgoing*, *camaraderie*), as well as emotionally positive (*beautiful*) and negative (*trouble*) words. These findings thus also support the potential for computerized natural language processing to contribute to understanding of the acute effects of psychoactive drugs like MDMA.

These initial analyses had several limitations: they focused on limited aspects of speech (semantic content), had small sample sizes, and did not use independent samples to test the classification algorithms developed. Here, we conducted a secondary analysis of a larger dataset to provide a more comprehensive assessment of natural language processing for detection of mental state changes during intoxication with MDMA (0.75; 1.5 mg/kg), compared to both placebo and intranasal oxytocin (20 IU). The larger study from which data were taken [19] investigated the social behavioral effects of oral MDMA compared to intranasal oxytocin, given that oxytocin administration produces some prosocial effects apparently similar to those of MDMA (e.g. see [20]). In addition to publication of the behavioral data from this study [19], a subset of these speech data were previously analyzed using the bag-of-words approach described above [18]. In this work, we perform a complete speech characterization using a broader range of features that includes semantic, acoustic, and psycholinguistic. We used a dataset composed of a description task (performed with minimal interaction with an interviewer) and a monologue task, as well as two independent datasets acquired in similar conditions for validation purposes. Within this setting, we aimed to test the following hypotheses: (i) each drug condition had a unique signature in speech, spanning different domains such as acoustics and content; (ii) the higher the dose of MDMA, the greater the associated changes in speech were; (iii) participants during the monologue could express their emotions more freely given the fact that they were alone in the room during the task; (iv) the trained models would generalize well in the independent validation datasets.

## Methods

### Participants

Healthy participants who reported using “ecstasy” or “molly” at least twice underwent comprehensive screening (medical examination, electrocardiogram, and structured interview) and provided written informed consent for participation. They then performed two different speech tasks after drug administration under procedures approved by the University of Chicago Institutional Review Board. Exclusion criteria included current medical illness or psychiatric disorder, body mass index outside of 18.5–30 kg/m^2^, cardiovascular disease, prior adverse ecstasy response, and pregnancy or lactation. Of 35 participants, 4 participants were discarded from the dataset since at least one of their 8 recordings (4 sessions × 2 speech tasks) were unusable. Two of them did not engage in the monologue task (they did not talk), while the other ones had very strong background noise in their recordings. Participants comprised 12 females (age 24.6 ± 4.7 years) and 19 males (24.1 ± 4.5 years). More information of the demographics and the substance at study entry can be found in Table 1.

**Table 1:** Demographics and substance use characteristics.

	Category	Training/validation dataset (N = 31)	ID1 (*N* = 36)	ID2 (*N* = 13)
Demographics	Sex	39% females	50% females	31% females
	Age	24.3 (4.4)	24.6 (4.7)	24.5 (5.4)
	Race	100% Caucasian	67% Caucasian, 11% African American, 3% Asian, 19% other/mixed race	84% Caucasian, 8% African American, 8% other/mixed race
	Education in years	14.7 (1.30)	15.1(1.5)	–
Current substance use	Alcohol drink per week	8.7 (6.8)	9.9 (10.6)	7.4 (5.5)
	Smoking past month	32%	22%	–
Lifetime occasions recreational use	MDMA	12.6 (9.3)	10.2 (8.2)	12.6 (19.1)
	Cannabis (days in past month)	7 (7.24)	64% (more than 100 times)	9.5 (10.8)

Notes: Statistically significant difference was found for race category when the training/validation set was compared to ID1 (*p*-value of 4E−4) and ID2 (*p*-value of 2E−2).

### Experimental protocol

The study employed a randomized, double-blind, within-between-participants design. All participants received placebo, two doses of MDMA (0.75 mg/kg and 1.5 mg/kg), and one dose of oxytocin (20 IU) over four different sessions. To account for potential differences in the time course of these drugs and facilitate blinding, a double-dummy approach was taken such that MDMA/placebo capsules were administered orally 30 min before an oxytocin/placebo intranasal spray. Thus, while participants received both a capsule and an intranasal spray in each session, they never received active MDMA and oxytocin together. Further information about drug doses and administration procedures has previously been published [18].

Sessions lasted from approximately 9 am until 1.30 pm and were spaced at least 5 days apart for drug washout. Before sessions, participants were asked to abstain from food for 2 h; cannabis for 7 days—if participants’ urine test was positive for cannabis, we followed up with a saliva test (Oratect, Branan Medical Corp., Irvine, CA); alcohol or medications for 24 h; and all other illicit drugs for 48 h. Compliance with these requirements was ascertained by urine (Ontrak TestStik, Roche Diagnostic Systems, Somerville, NJ), saliva (Oratect, Branan Medical Corp., Irvine, California), and breathalyzer (Alco-Sensor III Breathalyzer, Intoximeters, St Louis, MO) tests at the beginning of each session. Females were tested for pregnancy at each session. Speech tasks were conducted between approximately 75 and 105 min post-MDMA/placebo administration, coinciding with expected peak drug effects [21].

### Assessment measures

In each session, participants completed 2 speech tasks. The first, which we refer to as *Description*, comprised 5 min of free speech, with participants asked to talk about an important person in their life. The specific person was selected randomly each session from a list of four people provided previously by the participant. A research assistant listened, and if needed, they were trained to help the participant continue speaking by asking questions paraphrasing and reflecting the participant’s feelings. We have previously employed this approach to elicit free speech [16]. In the second task, *Monologue*, participants were asked to talk for up to 5 min (as much or as little as they liked) about any topic. A list of suggested topics was provided (e.g., family, friends, travel), but these were not limiting and participants could change topics as often as they wanted [22]. In the Monologue task, no listener was present. All speech was recorded using one channel at 44.1 kHz in WMA format. To analyze the semantic and syntactic speech content, a professional blind to drug condition manually transcribed the audio files.

### Analytic approach

#### Pre-processing

We extracted features based both on the acoustic properties of participants’ voices, and the information contained in transcripts. To ensure optimal reliability of the acoustic properties, the initial and final 30 s of each recording were not used for feature extraction. In addition, for the Description task, in which the research assistant was present and potentially speaking, his/her voice was manually removed from the recording. For the transcripts, in addition to removing the research assistant speech, we also removed punctuation and any special characters (e.g., #, $, [, etc.).

#### Feature extraction

To optimally mine the rich information in speech for mental state analysis, we extracted three feature types: acoustic, semantic, and psycholinguistic (i.e. syntactic). Below, details are provided about feature extraction (see also Table 2):

- *Acoustic features*: 88 acoustic features were extracted from each recording. The main software tools used for the feature extraction were Praat [23, 24] and Python (www.python.org). Features were extracted from five categories (see Table 2). First, we extracted features that characterize voice stability, including jitter, shimmer, and voice breaks. Then, noise was assessed with harmonic to noise ratio (HNR), noise to harmonics ratio (NHR), and mean autocorrelation. The third category includes temporal features such as the distribution of pauses and utterances. Pitch variations across the total recording time were also extracted. Information from the power spectrum is represented via Mel-frequency cepstral coefficients (MFCCs), which correlate with emotional states [25–27]. Finally, we extracted formant values, which characterize the acoustic resonance of uttered vowels in the vocal tract. Formant information is used to estimate the vowel space of each individual, which determines his/her vowel quality. Vowel space information reflects speaker characteristics, speech development, speaking style, sociolinguistic factors, and speech disorders (e.g. [28, 29]).
- *Semantic features*: To extract the semantic features, we employed a similar approach to that which we have previously used (LSA; see [16]). In the first stage, we processed transcripts with the Natural Language Toolkit (NLTK; [30]). Using the Treebank tagger in NTLK, we parsed interviews into sentences and identified nouns. Finally, we extracted the roots of words with the WordNetLemmatizer to obtain robust measurements. This generated a list of tokenized words for further processing. The second stage identified the semantic proximity between lemmatized words and several semantic concepts of interest by representing each word as a numeric vector based on its co-occurrence with every other word in a large corpus (the TASA corpus, a collection of educational materials compiled by Touchstone Applied Science Associates containing 7651 documents and 12,190,931 words, from a vocabulary of 77,998 distinct words). Using previous knowledge from MDMA research [31], we selected the following concepts of interest to best represent a range of subjective mental states likely impacted by MDMA: *affect, anxiety, compassion, confidence, disdain, emotion, empathy, fear, feeling, forgive, friend, happy, intimacy, love, pain, peace, rapport, sad, support, think*, and *talk*. A semantic proximity value was then calculated using cosine distance (dot product) between each concept of interest (using a unique word representation) and each word in the speech transcripts. Then, the median semantic proximity between each concept and the overall text was estimated. This procedure was repeated for the 21 concepts of interest, yielding 21 semantic features for each text.
- *Psycholinguistic features*: These features, capturing the lexical and syntactic complexity of speech, are divided into three categories. First, we used the Computerized Propositional Idea Density Rater (CPIDR [32]), to compute the total word count and number of ideas (expressed propositions) found in each transcript. Propositional density was also computed by dividing the number of ideas by the total word number. Second, we quantified parts of speech by dividing the number of occurrences of each part of speech by the total word number. This was done for pronouns, nouns, verbs, determiners, indefinites, and definites. Third, we extracted features to characterize participants’ lexical content. We used Honore’s statistic, a measure of lexical richness (number of words used exactly once) and Brunet’s index, also a measure of lexical diversity.

**Table 2:** Description of extracted speech features.

Type of Feature	Category	List of all features
Acoustic	Voice stability	Jitter, shimmer, voice breaks
	Noise measurements	Noise to harmonics ratio, harmonics to noise ratio, mean autocorrelation
	Pitch variations	Pitch distribution
	Spectral characterization	Max dB, max frequency, energy, slope
	Vowel space	Total area, ‘a-i-u’ area, Formants 1,2,3 distribution
	Mel-frequency cepstral coefficients (MFCC)	Sixteen MFCCs
	Temporal Features	Pause duration distribution, articulation and speech rates
Semantic	LSA (21 Concepts of interest)	*affect, anxiety, compassion, confidence, disdain, emotion, empathy, fear, feeling, forgive, friend, happy, intimacy, love, pain, peace, rapport, sad, support, think*, and *talk*.
Psycholinguistic	CPIDR	Ideas, total words, propositional density
	Parts of speech	pronouns, nouns, verbs, determiners, indefinites and definites, I (singular first person noun)
	Lexical content	Honore’s statistic and Brunet’s index, content words, total words, empty words, type-token, frequency, and fillers.

#### Condition-level comparisons

As a first step to analyze whether speech features differed between the placebo and active conditions, we performed a univariate analysis using paired Wilcoxon sign rank tests. Since we also wanted to evaluate the influence of the task on the extracted features, we performed these tests for each task separately. To correct for multiple comparisons, false discovery rate (FDR) correction at *q* < 0.05 was performed through the Benjamini–Hochberg procedure [33]. In addition, we analyzed the interactions between the features that pass FDR correction for all conditions using pairwise partial correlations, which measure the linear relationship between two variables while controlling for the effects of other variables. More specifically, partial correlations were calculated using the inverse of the regularized covariance matrix [34].

#### Classification

In addition to using condition-level descriptive analysis to evaluate if the extracted features were associated with mental states arising due to drug effects, we assessed their predictive performance through classification analysis. To detect the effects in the participants speech while they were under the influence of the analyzed drugs, we need to consider the inherent variability in speech across individuals. We illustrate this with the following example: some people talk faster than others. If a drug were to affect the speech rate of an individual by speeding it up and we observed its effect on a person that talks slowly, it is likely that this person would still talk slower than a person that usually talks very fast. Therefore, the effect of the drug would remain unnoticed. For this reason, we decided to adjust for these differences by correcting the speech characteristics of each individual by their own baselines. By doing so, we would effectively measure the differential effect of a drug in each individual. We followed this rational to detect the effects of a drug with respect to placebo by subtracting their feature representations. On the other hand, if we wanted to explore the effect of placebo with respect to the drug, we would need to reverse the sign of the subtraction. In other terms, classifying condition A vs B is equivalent to classifying (A – B) vs (B – A). More details of this approach can be found in [16]. After generating features based on this representation, we evaluated the following classification tasks: placebo vs. each active drug condition (MDMA 0.75 mg/kg; MDMA 1.5 mg/kg, and oxytocin), and MDMA 0.75 mg/kg vs. MDMA 1.5 mg/kg for the *Description* and *Monologue* tasks individually. Prior to classification, all features were standardized to a mean of 0 and standard deviation of 1. We employed three classifier types to evaluate the predictive power of speech features to differentiate between conditions: (a) linear support vector machines (SVM), which estimates based on linear combinations of features; (b) nearest neighbors, whose predictions are based on similarity metrics between samples; and (c) a non-linear classifier based on decision trees called random forest. To identify performance of the classifiers and optimize the parameters, we used a nested leave-one-participant-out cross-validation approach. Finally, since both group representations come from both sessions of the same set of subjects, the probability of detecting either condition by chance is exactly 50%. To perform feature selection, we ranked the features using two sample t-tests with samples of the training set as we were measuring changes (as opposed to absolute values) associated with drug effects. We report the cross-validation performance obtained using the optimal set of features.

#### Multivariate analysis

As a post hoc analysis, we checked the weights obtained by the best models generated for the different classification tasks. Since the contribution of the most informative features was evaluated in terms of weights assigned by the classifier, this could only be achieved through the analysis of the linear classifier: linear SVM. To be able to compare the weights assigned across models, these were rescaled to the range of 0 to 1 by (1) taking their absolute values, and (2) dividing them by their sum across all features. By doing so, we had the contribution of each feature to the classification as a percentage value. To reduce the complexity of this depiction, we only focus on the features that had a relative contribution of more than 10%. It should be noted that since these are the results of a cross-validated approach, different sets of optimal are found across folds.

#### Validation

Models were estimated on the training dataset (*N* = 31) described above. We then validated the models in two independent datasets in which participants had also undergone the *Description* task after MDMA (0.75, 1.5 mg/kg) and placebo. The same set of features described above was extracted from these independent datasets, with the exception of acoustic data, which was not available due to lower quality audio recordings. In addition, in one of these datasets (Independent Dataset 2; ID2), the duration of the task was 10 instead of 5 min, so two psycholinguistic features that vary with task duration (total word count and number of ideas) were not considered for model validation in ID2. Independent Dataset 1 (ID1) comprised data from 36 healthy participants (18 females; age = 24.6 ± 4.7 years) who completed a 3-session within-participants study, receiving placebo, low dose MDMA (0.75 mg/kg) and MDMA (1.5 mg/kg). Further details of the overall study from which ID1 was obtained are described in a previous publication, which employed a word count method to assay positive and negative words used in the speech task [35]. The speech data in ID1 were collected 140 min after MDMA/placebo administration. ID2 was comprised of data we previously analyzed for semantic and syntactic features of speech [16]. This dataset is composed of speech data from 13 participants (4 females; overall age = 24.5 ± 5.4 years) who also completed a within-participants study and received placebo and MDMA (0.75, 1.5 mg/kg) across 3 sessions; details of this study have previously been reported ([16, 36] participants also underwent a methamphetamine session in ID2, however these data are not included in this analysis). Speech data for ID2 were collected 130 min post MDMA/placebo administration. Demographic information of ID1 and ID2 is provided in Table 1. The only variable that presents statistically significant differences between the train/validation dataset and the independent datasets is race, showing *p*-values of 4E–4 (train/validation set vs ID1) and 2E–2 (train/validation set vs ID2) for the proportion of Caucasian individuals.

## Results

### Condition-level comparisons

The top three features (identified by the lowest *p*-value) for each of the four comparisons (e.g., MDMA 0.75 vs. placebo) by speech task (i.e. Description and Monologue) are presented in Table 3. Ten features were found to show statistically significant differences across conditions after FDR correction. Acoustic features appear to be more relevant to detect the effects of oxytocin. In addition, different sets of relevant features were observed for the two different speech tasks.

**Table 3:** Univariate analysis: features ranked using the *p*-value of Wilcoxon paired *t*-test.

Conditions	Monologues feature name			Description feature name		
	Acoustic	Semantic	Psycholinguistic	Acoustic	Semantic	Psycholinguistic
MDMA 0.75 vs. PBO	Pitch_a_	Think*	W-Empty	F1_b_	Sad*	Density
	Pitch_e_	Talk*	Determiners	Angle	Happy	W-Empty
	MFCC #13_b_	Feeling*	Indefinites	PauseDist_e_	Confidence	N-Nouns
MDMA 1.5 vs. PBO	MFCC #12_a_	Talk	Frequency	Angle	Support	Ideas*
	F3_e_	Love	Determiners	PauseDist_a_	Think	Honores*
	PauseDist_g_	Peace	Definites	PauseDist_b_	Love	W – Total*
MDMA 0.75 vs. MDMA1.5	Pitch_a_	Support	Frequency	PauseDist_a_	Sad	W-Empty *
	MFCC #4_b_	Think	Determiners	PauseDist_g_	Love	W-Total
	MFCC #12_a_	Affect	Density	PauseDist_b_	Rapport	W-content
OT vs. PBO	F2_c_*	Emotion	Density	PauseDist_a_	Support	Definites
	F2_b_*	Anxiety	N-Nouns	Shimmer_h_	Peace	Determiners
	F2_e_	Talk	N-Verbs	Unvoiced_i_	Feeling	W-empty

Notes: Sub-index in the name of the feature indicate the descriptor: (a) median, (b) IQR, (c) kurtosis, (d) skewness, (e) percentile 5th, (f) percentile 50, (g) percentile 95th, (h) local; (i) frames. W refers to number of words. * indicates that the test passed FDR correction. PBO = placebo; MDMA 0.75 = 3,4-methylenedioxymethamphetamine 0.75 mg/kg; MDMA 1.5 = 3,4-methylenedioxymethamphetamine 1.5 mg/kg; OT = oxytocin 20 international units.

### Partial correlations

**Fig. 1:**
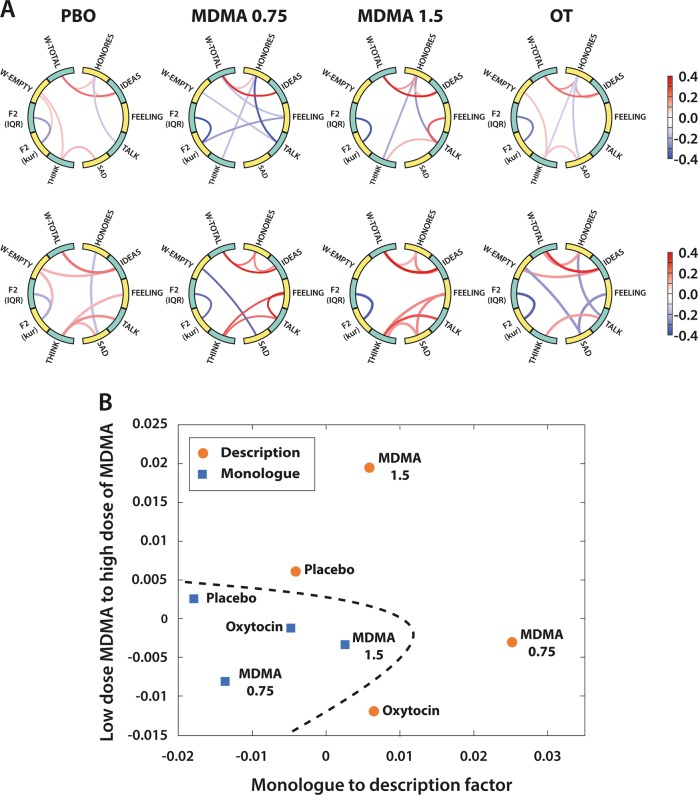
**a** Partial correlations between the statistically significant features found in Table 2 identified as a function of drug condition and task (Monologue presented in the top row and Description in the bottom row). **b** Multidimensional scaling representation of the partial correlations in Fig. 1a. Observe the horizontal axis differentiating the monologue and description task for each drug condition, and the vertical axis differentiating low and high MDMA conditions for each task. Moreover, the dashed line contains exclusively all of the monologue tasks, stressing the consistency of the representation with the experimental conditions.

Partial correlations were conducted to examine the relationships between pairs of features that best differentiated conditions (see Table 3). Figure 1a presents the structure and strength of partial correlations among these features as a function of condition (columns) and task type (rows), while Fig. 1b provides a multidimensional mapping of the partial correlations shown in Fig. 1a. Stronger associations were found when the subjects were under the influence of psychoactive drugs relative to placebo, especially MDMA. The projection of the partial correlations in two dimensions show that each speech task has roughly a different location along one of the axis of this subspace, in this subspace and that, regardless of the speech task, the increased dose of MDMA can be detected by the second dimension in this subspace (axis y in Fig. 1b).

### Classification

**Fig. 2:**
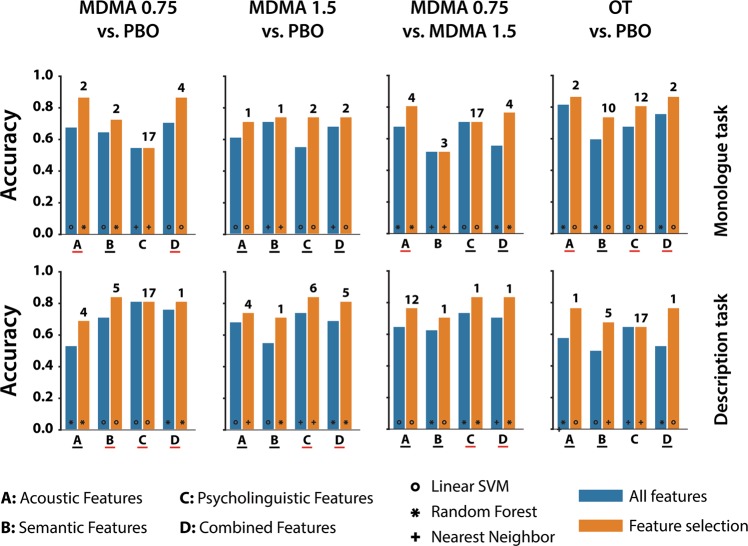
Classification accuracy by task, feature type, and binary condition comparison. The number of features obtained after feature selection is specified at the top of each bar. The symbols at the bottom of the bar indicate with which algorithm the maximum accuracy was achieved: o Linear SVM, * Random Forest, and + Nearest neighbors. The types of features are indicated as follows: A = Acoustic features only; B = Semantic features only; C = Psycholinguistic/syntactic features only; D = Combined features. PBO = placebo; MDMA 0.75 = 3,4-methylenedioxymethamphetamine 0.75 mg/kg; MDMA 1.5 = 3,4-methylenedioxymethamphetamine 1.5 mg/kg; OT = oxytocin 20 international units. Letters underlined in black indicate that at least one of the models achieved classification higher than chance at p < 0.05, underlined in red at *p* < 0.001.

The accuracy of the four binary classifications (cross-validated) in the Monologue and Descriptions task are presented in Fig. 2. Classification using acoustic features only is more accurate for the Monologue than the Description task. Conversely, features obtained from transcripts (semantic and psycholinguistic) were more informative for the Descriptions task. The highest accuracy observed was for classification of a low MDMA dose relative to placebo, where features extracted from speech yielded accuracy of up to 87 and 84% with feature selection for Monologue and Description tasks, respectively. The entire set of features did not always improve classification accuracy. Regarding the use of classifiers, the most accurate classifications were obtained using linear SVM, followed by Random Forest and Nearest Neighbors. We implemented a binomial test to estimate significance of the prediction accuracy.

### Multivariate analysis

**Fig. 3:**
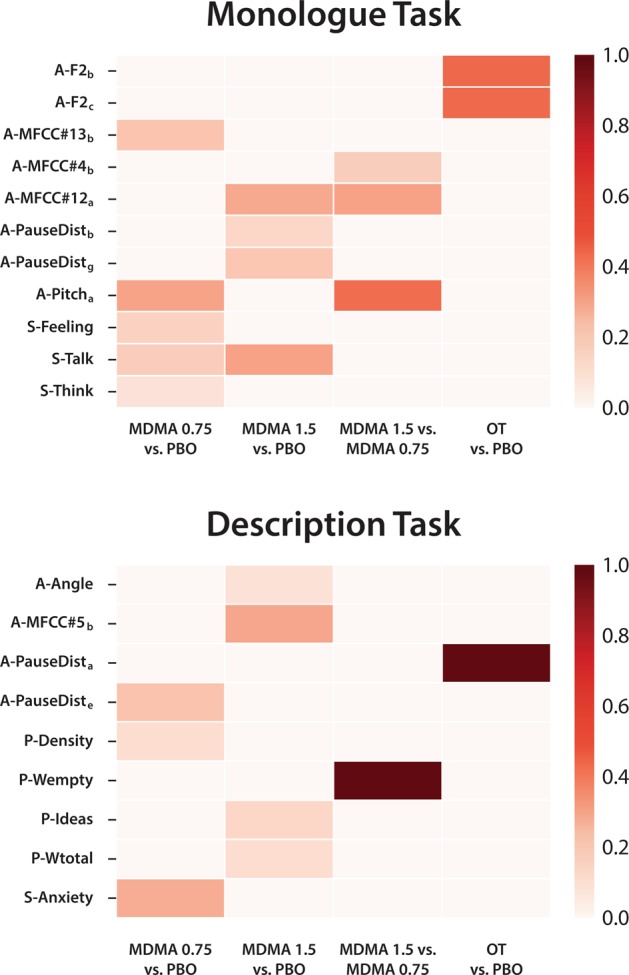
Weight representation of combined features found by optimal linear classification models (2 tasks x 4 conditions). Weights are normalized to represent the relevant contribution of each feature as percentages. Two heatmaps are shown corresponding to both speech tasks analyzed in this study (left: monologue, right: description). Features that contributed less than 10% were not displayed here. First letter in the feature name indicates the type of feature: A = Acoustic, S = semantic, P = Psycholinguistic.

Weight contributions in each of the linear optimal models is shown in Fig. 3. Although linear SVM models achieved the highest accuracy for only three out of the eight analyzed conditions (See Fig. 2, combined features + features selection), the accuracies of all of the analyzed linear SVM models are good, with results in the range of [71% 87%]. We observe that for the monologue task, psycholinguistic features do not have any contribution to either of the four classification tasks. However, for the description task, psycholinguistic features appear to be relevant for three of the four classification tasks. We also observe that most of the top features reported in the univariate test (Table 3) are also relevant for classification, except for MDMA 0.75 vs. PBO, in which *anxiety* appears to have a predominant role relative to the semantic features reported in Table 3.

### Validation

**Fig. 4:**
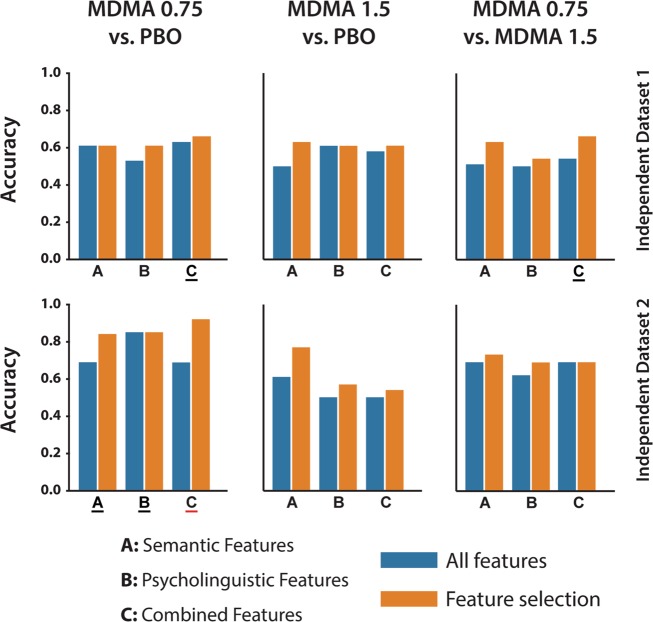
Classification accuracy by feature type and binary condition comparison in the validation datasets. PBO = placebo; MDMA 0.75 = 3,4-methylenedioxymethamphetamine 0.75 mg/kg; MDMA 1.5 = 3,4-methylenedioxymethamphetamine 1.5 mg/kg. Letters underlined in black indicate that at least one of the models achieved classification higher than chance at *p* < 0.05, underlined in red at *p* < 0.002 (note these are pessimistic significance estimates based on multiple differences between the training and validation datasets).

The accuracy of the classifiers generated on the training data was tested in two separate validation datasets (Description task only) for three of the four conditions (Fig. 4). Accuracy values up to 92 and 66% were achieved for ID1 and ID2, respectively (chance = 50%) using models enriched with feature selection. We also implemented a binomial test for significance; however, this is a pessimistic measure of significance for a validation dataset that, as is the case here, differs from the training dataset in several experimental dimensions [37, 38]. Even with this conservative approach, we observed discrimination accuracies significantly higher than chance.

## Discussion

These analyses represent, to the best of our knowledge, the first attempt to use a broad spectrum of speech characteristics to assess acute mental state changes as a result of drug intoxication in a laboratory setting. Previous studies investigating the acute effects of drugs on speech have employed a range of different analytic methods, including automated semantic and syntactic analysis [16, 18], computerized word count methods [35], and manual approaches (e.g. [39, 40]). The results presented here suggest that a broad array of computer-extracted speech features, including acoustic, semantic, and psycholinguistic variables, can provide a more complete characterization of all speech changes generated by the acute effects of MDMA and intranasal oxytocin administration. Indeed, the complexity of these results highlights both the richness of speech as a data source and the difficulty inherent in identifying which features are most important in relation to specific drug effects.

We found that (1) The top features identified in the analysis were related to both the drug and the task employed (i.e. whether it was a description elicited via questions from a researcher or a monologue); (2) Within each drug condition, associations (partial correlations) between speech variables varied with the task; (3) Accuracy varied with drug, feature type, and task type; (4) Higher dose of MDMA is not associated with higher changes with respect to placebo; and (5) Combining features in machine learning classifiers consistently yielded accuracy rates higher than chance, including when tested in two independent datasets. These data indicate that speech analysis shows promise as an assay of acute drug effects, providing further proof-of-concept evidence for computerized use of speech to measure mental states in humans. For example, automated speech analysis could potentially aid psychiatry to overcome the limitations of traditional assessments, such as compensate for the limited number of trained professionals to evaluate various aspects of communication or aid them to monitor changes over time [8].

Several aspects of these results warrant comment. In condition-wise comparisons, features differing between conditions varied both with task and drug comparison. For example, in the oxytocin vs. placebo comparison, the top features identified passed FDR correction for the Monologue, but not the Description task. The top acoustic features in the Monologue task across comparisons were related to MFCCs and formants, which are known to reflect affective states [41]. For example, mean MFCC values differentiate between boredom and neutral emotions [42]. The position of the formants in the vowel space has also been studied for emotion recognition from speech [43], finding that formant frequency values reflect speech valence. Specifically, positive emotions and high arousal are associated with higher second formant (F2) values, which we also observed under oxytocin compared to placebo in the Monologue task. In fact, weight analysis revealed that a very high accuracy was achieved with only F2 features (See Fig. 3). Conversely, the top acoustic features in the Description task were related to the duration of pauses during speech. This difference may suggest that our hypothesis actually holds and the presence of the research assistant during the Description task prevented participants from expressing their emotions as freely as they could in the Monologue. Alternatively, this may reflect the different instructions given for each task. Either possibility indicates that the optimal conditions for eliciting speech for the purpose of automated speech analysis represent a critical factor in need of further research.

We can provide an illustrative template for interpreting how the mental states underlying the eight conditions (four drugs x two tasks) determine interactions between speech components. Figure 1a shows partial correlations between the most relevant features identified. Across tasks, partial correlations were low, with more pronounced strength in the active drug conditions. In these, it is interesting to note that the only substantial link between an acoustic feature (F2 kurtosis) and a linguistic feature (“feeling”) is observed for the monologue with low dose MDMA. Similarly, the description task with a high dose of MDMA is the only condition that uncouples structural linguistic features (w-empty, w-total, Honoré’s and ideas) from semantic features (“think”, “sad”, “talk”, “feeling”). Further insights can be obtained by mapping the partial correlation matrices onto a two-dimensional space using Multidimensional Scaling (MDS), which arranges them according to their similarity [44]. MDS (Fig. 1b) finds that the two main dimensions of similarity are one that is determined by the task (“monologue to description factor”), such that for each drug condition *description* instance is to the right of *monologue*, and another dimension that determines MDMA dose level, such that for both tasks *high MDMA* is higher than *low MDMA*. Although the effect of a higher doses of MDMA is in the same direction regardless of the tasks, we observe that a higher dose of MDMA does not yield to a higher distance from placebo, as we hypothesized at the beginning of this work. This is also corroborated by the performance of our models where a slightly higher performance is obtained for MDMA 0.75 vs. placebo than for MDMA 1.5 vs. placebo.

The overall accuracy of cross-validated classification, which was higher than chance, was consistent with previous findings by our group indicating that, at least in cross-validation, automated speech analysis can contribute to higher-than-chance classification between drug conditions [16]. Here, we extended significantly on those findings to report that speech-based classifiers trained on one dataset yield higher-than chance classification in independent validation datasets (see Fig. 4). These results also, however, highlighted the impact of methodological details, with classification accuracy higher in Independent Dataset 2 relative to Independent Dataset 1. One possible factor in this difference is that the speech task in ID2 (130 min after dosing) was conducted closer to peak drug effects and the time of the speech task in the training dataset (between 75 and 105 min) than that in ID1 (140 min after dosing). Another possible factor is that for ID1, there is a statistically significant difference (*p*-value = 4E–4) with the training/validation dataset in terms of the proportion of Caucasian participants. We speculate that different races, which may be associated with different cultural backgrounds, could have an effect in how people perform the description task that we were not able to account for. This suggests a further axis of complexity in characterizing drug effects via automated speech, with features and classification accuracy varying along the time-course of drug effects as well as with drug, dose, speech elicitation task, and potentially race or ethnicity.

As a secondary analysis, this study has several limitations. First, the three datasets employed for analyses were designed for other studies [19, 36, 45, 46]. Moreover, subsets of the data have been used in previous analyses of speech features [16, 18, 35]. However, the current analyses significantly extended prior findings by: (1) Including acoustic, semantic, and psycholinguistic features; and (2) Training classifiers on one dataset and testing them on two independent datasets. Second, because the original studies were not designed for the present purpose, there were methodological differences between studies including variation in the position of the speech task in the drug time course, and differences in the specific tasks used. While not optimal, this variability did point towards important factors potentially influencing outcome for further empirical investigation. Third, we only assessed a limited number of drug conditions (two doses of MDMA, and one of oxytocin for each participant). An obvious extension of these findings would be to investigate the potential capacity of computerized speech analysis to detect intoxication with more commonly used drugs. This is particularly relevant for cannabis, given that biochemical markers may not provide a reliable test of impairment (rather than exposure), which is increasingly necessary in the context of legalization of cannabis for medicinal and recreational purposes [47, 48].

These findings contribute to a small but rapidly growing body of literature suggesting that computerized speech analysis methods may present a powerful, non-invasive, and cost-effective way to capture clinically relevant mental states, including those occurring during intoxication. Further work is needed to refine these methods and reduce the complexity of speech data mining into usable algorithms; in particular, larger and more varied datasets would help considerably to identify which speech markers are independent of the particular task and experimental setting, and also allow for a systematic exploration of interpretable data-driven markers [49]. However, these methods suggest that in the near future, digital phenotyping, including automated speech analysis, could provide reliable, objective information to complement existing methods used to understand human mental states.

## Funding and disclosure

This research was supported by the National Institute on Drug Abuse (DA026570, DA02812, DA040855, DA034877). The authors have no conflicts of interest to declare.
